# Overexpression of *HIPK2* removes the transrepression of proapoptotic genes mediated by the CtBP1-p300-FOXO3a complex and increases the chemosensitivity in osteosarcoma cells

**DOI:** 10.7150/jca.52115

**Published:** 2021-01-21

**Authors:** Ning Duan, Wentao Zhang, Zhong Li, Liang Sun, Tao Song, Zirui Yu, Xun Chen, Wei Ma

**Affiliations:** 1 Department of Orthopedics, the First Affiliated Hospital of Xi'an Jiaotong University, Xi'an, Shaanxi, 710061, China.; 2 Department of Orthopedic Surgery, Honghui Hospital, Xi'an Jiaotong University, Xi'an, Shaanxi, 710054, China.

**Keywords:** CtBP1, HIPK2, proapoptotic genes, chemosensitivity, osteosarcoma

## Abstract

Decreased expression of proapoptotic genes can lead to the chemoresistenance in cancer therapy. Carboxyl-terminal binding protein 1 (CtBP1), a transcriptional corepressor with multiple oncogenic effects, has been previously identified to suppress the expression of two proapoptotic genes [*BAX* (BCL2 associated X) and *BIM* (Bcl-2 interacting mediator of cell death)] by assembling a complex with the Forkhead box O3 (FOXO3a) transcription factor and the p300 histone acetyltransferase. However, the upstream regulatory signaling of the CtBP1-p300-FOXO3a complex is obscure, and the effects of changing this signaling on chemosensitivity in osteosarcoma are unknown. Herein, we discovered that the downregulation of *HIPK2* (Homeodomain-interacting protein kinase 2) was essential for the function of the CtBP1-p300-FOXO3a complex. Downregulation of *HIPK2* prevented the phosphorylation and subsequent degradation of CtBP1, thereby allowing the assembly of the CtBP1-p300-FOXO3a complex and suppression of the expression of proapoptotic genes, such as *BAX*, *BIM*, *BIK* (Bcl-2 interacting killer) and *NOXA*/*PMAIP1* (Phorbol-12-myristate-13-acetate-induced protein 1). Overexpression of *HIPK2* promoted the phosphorylation of CtBP1 and the degradation of CtBP1 by proteasomes, thereby preventing the formation of the CtBP1-p300-FOXO3a complex. The abolition of CtBP1 transrepression increased the expression of proapoptotic genes to induce apoptosis and increase chemosensitivity in osteosarcoma cells. Taken together, our *in vitro* and *in vivo* results revealed that overexpression of *HIPK2* could remove the CtBP1-mediated transrepression of proapoptotic genes, indicating a new therapeutic option for the treatment of osteosarcoma.

## Introduction

The triggering of apoptosis is a major mode of many chemotherapeutic drugs used to eliminate cancer cells 1-3. The signaling pathways that mediate apoptosis are therefore a key focus in the development of chemotherapeutic drugs 1-3. Increasing evidence now indicates that the blockage of apoptotic signaling is associated with cancer chemoresistance 1-3. Two apoptotic pathways, the extrinsic and the intrinsic pathways, are involved in the activation of caspases, a representative symptom of apoptosis 4. The extrinsic pathway is initiated by the activation of tumor necrosis factor receptors (TNFRs) on the cell membrane and triggers a cascade of caspases, including caspase-8 and caspase-10 4. By contrast, the intrinsic pathway is activated by the cellular stress imposed by deprivation of growth factors, exposure to cytotoxic chemicals, or agents that damage DNA 4. A critical step in the intrinsic pathway is the release of cytochrome *c* from the mitochondrial intermembrane space 5,6. The integrity of the mitochondrial membrane is controlled by the BCL2 (B-cell lymphoma 2) family proteins, including the pro-survival members [BCL2, BCL-xL, BCL-w and MCL1 (Myeloid cell leukemia 1)] and proapoptotic members [BAX (BCL2 associated X), BAK1 (BCL2 antagonist/killer 1), BIM (Bcl-2 interacting mediator of cell death), BIK (Bcl-2 interacting killer) and NOXA/PMAIP1 (Phorbol-12-myristate-13-acetate-induced protein 1), PUMA (P53 up-regulated modulator of apoptosis), and BID (BH3 interacting domain death agonist)] 5,6. The pro-survival proteins inhibit the release of cytochrome *c*, while overexpression of proapoptotic proteins promotes the permeabilization of the mitochondria 5,6. In addition to their activation by cellular stresses, the proapoptotic proteins are also regulated by transcription factors 5,6. In osteosarcoma cells, the expression levels of *BAX* and *BIM* levels can be suppressed by a transcriptional complex assembled by a corepressor CtBP1 (Carboxyl-terminal binding protein 1), the p300 histone acetyltransferase, and the Forkhead box O3 (FOXO3a) transcription factor 7. The expression of *PUMA* is controlled by different transcription factors, such as p53 8, FOXO3a 9, and SOX4 10. *BAK* is regulated by p53 and p73 11.

The human and mouse genomes encode two CtBP homologues known as CtBP1 and CtBP2, which share nearly 80% amino acid identity 12. CtBP1 is highly expressed during early embryonic development but it remains silent at other developmental stages 13. In many cancers, however, CtBP1 is reactivated and its overexpression can promote cell proliferation and genomic instability 14,15, inhibit apoptosis 7, and induce the epithelial-mesenchymal transition (EMT) 16. CtBP1 promotes cell survival but blocks apoptosis by suppressing the expression of several proapoptotic genes, including *BAX*, *BIM*, *BIK1*, *NOXA*, and *PUMA* 12. Overexpression of *CtBP1* also affects genome instability by inhibiting the expression of *BRCA1* (Breast cancer 1) and *BRCA2* 12. CtBP1 induces the EMT process through repression of *CDH1* (Cadherin 1, also known as E-cadherin), an epithelial marker 17. Mechanically, CtBP1 couples with transcription factors and other transcriptional regulators to assemble into different complexes that specifically bind to the promoter regions of genes to control gene expression 7. For example, in osteosarcoma cells, CtBP1 is overexpressed and the resulting CtBP1-p300-FOXO3a complex controls the expression of two proapoptotic genes* BAX* and *BIM* 7. However, the upstream signaling of the CtBP1-p300-FOXO3a complex has not been identified.

Previous studies have shown that CtBP1 can be phosphorylated by homeodomain-interacting protein kinase-2 (HIPK2) in cells exposed to UV radiation and that the phosphorylated CtBP1 is degraded through the proteasomal pathway 18. HIPK2 is a serine/threonine kinase that controls cell proliferation, DNA repair, cellular senescence and cell death 19. In the process of tumorigenesis, HIPK2 functions as a tumor suppressor and its expression is significantly downregulated in several cancer types, such as thyroid carcinomas, skin cancer, and breast cancer 19. However, the expression status of HIPK2 in osteosarcoma cells is not known, and a role for HIPK2 in the phosphorylation of CtBP1 and tumorigenesis remains to be established in osteosarcoma.

In the present study, we investigated the role of HIPK2 in the tumorigenesis of osteosarcoma by determining its expression level in osteosarcoma cells and biopsies. We observed a significant decrease in the expression levels of HIPK2 and phosphorylated CtBP1 in both osteosarcoma cells and biopsies and an opposite response in the CtBP1 expression level. Overexpression of *HIPK2* reversed *CtBP1* expression, induce apoptosis and increase chemosensitivity to anticancer drugs both *in vitro* and *in vivo*. Our results provide a new avenue for increasing the chemosensitivity of osteosarcoma cells.

## Materials and methods

### Vector constructions

The coding sequences of *CtBP1* and *HIPK2* genes were cloned into empty pCDNA3 vectors using the BamHI and XhoI sites. The wild type (WT) promoters (2,000 bp) of *BIM*, *BIK*, *BAX*, and *NOXA* genes were respectively cloned into empty pGL4.26 luciferase vectors using the SacI and XhoI sites. The generated pGL4.26-pBIM^WT^, pGL4.26-pBIK^WT^, pGL4.26-pBAX^WT^, and pGL4.26-pNOXA^WT^ plasmids were used as templates to create their corresponding mutants in which the FOXO3a consensus site (A/G)TAAA(T/C)A was mutated to CAGGGAG. The primers used for vector constructions were included in Supplementary Table 1. All plasmids were sequenced to verify their correct constructions.

### Cell culture and transfection

The hFOB1.19 (#CRL-11372) human osteoblast cell line and the DAN (#CRL-2130), MG63 (#CRL-1427), HOS (#CRL-1543), T1-73 (#CRL-7943), 143B (#CRL-8303), Saos2 (#HTB-85), and U2OS (#HTB-96) osteosarcoma cell lines were purchased from American Type Culture Collection (ATCC) (Manassas, VA, USA). All cell lines were cultured in Dulbecco's Modified Eagle's Medium (DMEM) (Corning, USA, #10-017-CM) containing 10% fetal bovine serum (FBS) (Sigma-Aldrich, China, #F2442) and 100 units/mL of penicillin-streptomycin (Sigma-Aldrich, #P4333). All osteosarcoma cell lines were grown in a 37 °C incubator, and the hFOB1.19 cells were cultured at 34 °C. For cell transfection with shRNAs, the lentiviral transduction particles of CtBP1 (Sigma-Aldrich, #TRCN0000285086) and HIPK2 (Sigma-Aldrich, # TRCN0000433047) and one control particle containing pLKO.1 empty vector were transfected into cells using FuGene 6 (Roche Diagnostics Corp., USA, #E2691) following the manufacturer's protocol. The transfected cells were allowed to recover for 12 h and then selected with 1 μg/mL puromycin (Sigma-Aldrich, #540411). Single puromycin-resistant cells were collected and used to confirm gene expression. Cells showing successful knockdown of *CtBP1* and *HIPK2* were used for subsequent experiments. For cell transfection with plasmids, the pCDNA3-HIPK2 and pCDNA3 empty vectors were individually transfected into cells with the Lipofectamine 3000 reagent (Thermo Fisher Scientific, China, #L3000015). Cells showing successful transfection of HIPK2 were used in subsequent experiments.

### Total RNA extraction and real-time quantitative PCR (RT-qPCR) analysis

Total RNA was extracted from cells and biopsies using an RNeasy Plus Kit (Qiagen, Germany, #74134) following the manufacturer's protocol. The concentrations of RNA samples were quantified with a Nanodrop instrument (Thermo Fisher Scientific, #ND-2000) and 1.0 µg total RNA was reversely transcribed with a PrimeScript RT reagent kit (Takara, China, #RR0378) to produce cDNA. The obtained cDNA samples were diluted 20-fold, followed by RT-qPCR analyses with a SYBR Green quantitative RT-qPCR kit (Sigma-Aldrich, #QR0100). The primers were listed in Supplementary Table 2. The relative expression levels of different genes were normalized to β-Actin according to the 2^-∆∆Ct^ method.

### Western blotting

Western blotting analysis was performed following a previously described method 20. Briefly, cells were lysed in radioimmunoprecipitation assay (RIPA) buffer (Sigma-Aldrich, #R0278) containing protease inhibitor (Sigma-Aldrich, P8340). Equal amounts of total proteins were loaded and resolved by SDS-PAGE gels, followed by transferring to a PVDF membrane, blocking with 5% milk, and probing with primary antibodies. The following primary antibodies were used: anti-CtBP1 (BD Bioscience, USA, #612042), anti-CtBP1 (phospho Ser422) (GeneTex, USA, #GTX55356), anti-CtBP2 (BD Bioscience, #612044), anti-HIPK2 (Cell Signaling, USA, #5091S), anti-BIM (Abcam, China, #ab170589), anti-BIK (Abcam, #ab52182), anti-BAX (Abcam, #ab3191), anti-NOXA (Abcam, #114C307), anti-CASP3 (Sigma-Aldrich, #C9598), anti-CASP7 (Sigma-Aldrich, #C1104), anti-CASP9 (Abcam, #ab184786), anti-p300 (Sigma-Aldrich, #P2859), anti-FOXO3a (Sigma-Aldrich, #V38041), and anti-GAPDH (Thermo Fisher Scientific, #MA5-15738-BTIN). The protein signals were visualized using an ECL detection kit (Sigma-Aldrich, #GERPN2109).

### Immunoprecipitation

Total cell lysates from MG63 cells were immunoprecipitated using anti-CtBP1- and IgG-coupled protein A beads (Sigma-Aldrich, #16-125), respectively. Cell lysates and beads were incubated at 4 °C overnight, followed by washing five times with RIPA buffer. The input proteins and the purified CtBP1-coupled and IgG-coupled proteins were subjected to western blotting to examine the protein levels of CtBP1, p300, and FOXO3a, respectively.

### *In vitro* oncogenic phenotype assays

The oncogenic phenotype assays, including cell proliferation, colony formation, and cell invasion, were performed as described previously 21. For cell proliferation, equal numbers of cells (~1000) were seeded into 96-well plates (Thermo Fisher Scientific, #08-772-2C), and cell viability was determined with an MTT kit (Sigma-Aldrich, #114650007001) at 1-day interval for five days. For cell colony formation assay, equal numbers of cells (~500) were seeded into 6-well plates and grown at 37 °C for 14 days with medium renewal every three days. After fixing with 4% paraformaldehyde (Sigma-Aldrich, #P6148), colonies were stained with 0.1% crystal violet (Sigma-Aldrich, #C0775). For cell invasion assay, equal amounts of cells (~500) were suspended into DMEM without FBS and seeded into the upper Boyden chamber, the lower chambers chamber contained DMEM with 10% FBS. The whole Boyden chambers were placed at 37 °C overnight. Cells that invaded into the lower chambers were fixed with 4% paraformaldehyde, stained with 0.1% crystal violet, and photographed with a microscope (Nikon, Japan, #SMZ800).

### Luciferase assay

The pGL4.26-pBIM^WT^, pGL4.26-pBIK^WT^, pGL4.26-pBAX^WT^, pGL4.26-pNOXA^WT^, and their corresponding mutant luciferase vectors were co-transfected with Renilla (internal control) into the Control-KD (knockdown), FOXO3a-KD, Control-OE (overexpression), and FOXO3a-OE cells, respectively. After an 18 h recovery at 37 °C, the cells were used for luciferase assay with a Dual Luciferase Reporter Assay System (Promega, USA, #E1910). The relative luciferase activity was normalized using firefly luciferase activity/Renilla luciferase activity.

### Tumor xenograft model

Eight-week-old C57BL/6 mice with similar weight (approximately 24 g) were intradermally injected with the following cell suspensions: Control-OE in MG63 background (MG63), MG63 expressing pCDNA3-HIPK2 (MG63+HIPK2), Control-OE in Saos2 background (Saos2), and Saos2 expressing pCDNA3-HIPK2 (Saos2+HIPK2), respectively. Tumor volumes were determined at 5-day intervals with the following formula: volume = (length × width^2^)/2. After 25 days, mice with similar tumor volumes (approximately 200 mm^3^) in each group were divided into three groups (n=10 for each group) and injected with PBS, cisplatin (CDDP), or methotrexate (MTX). Tumor volumes were determined at 5-day intervals for another 30 days. All experimental procedures were performed according to a protocol reviewed and approved by the Institutional Animal Care and Use Committee (IACUC) of the First Affiliated Hospital of the Medical College in Xi'an Jiao Tong University.

### Statistical analysis

All experiments in this study were independently repeated at least three times. The experimental data were analyzed with SPSS 26 software and were displayed as the mean ± standard deviation (SD). Statistical significance was set at* P* < 0.05 (*), *P* < 0.01 (**) and *P* < 0.001 (***) according to the results of two-sided Student's t test.

## Results

### *CtBP1* and *HIPK2* showed inverse expression levels in osteosarcoma cells and biopsies

**Figure 1:**
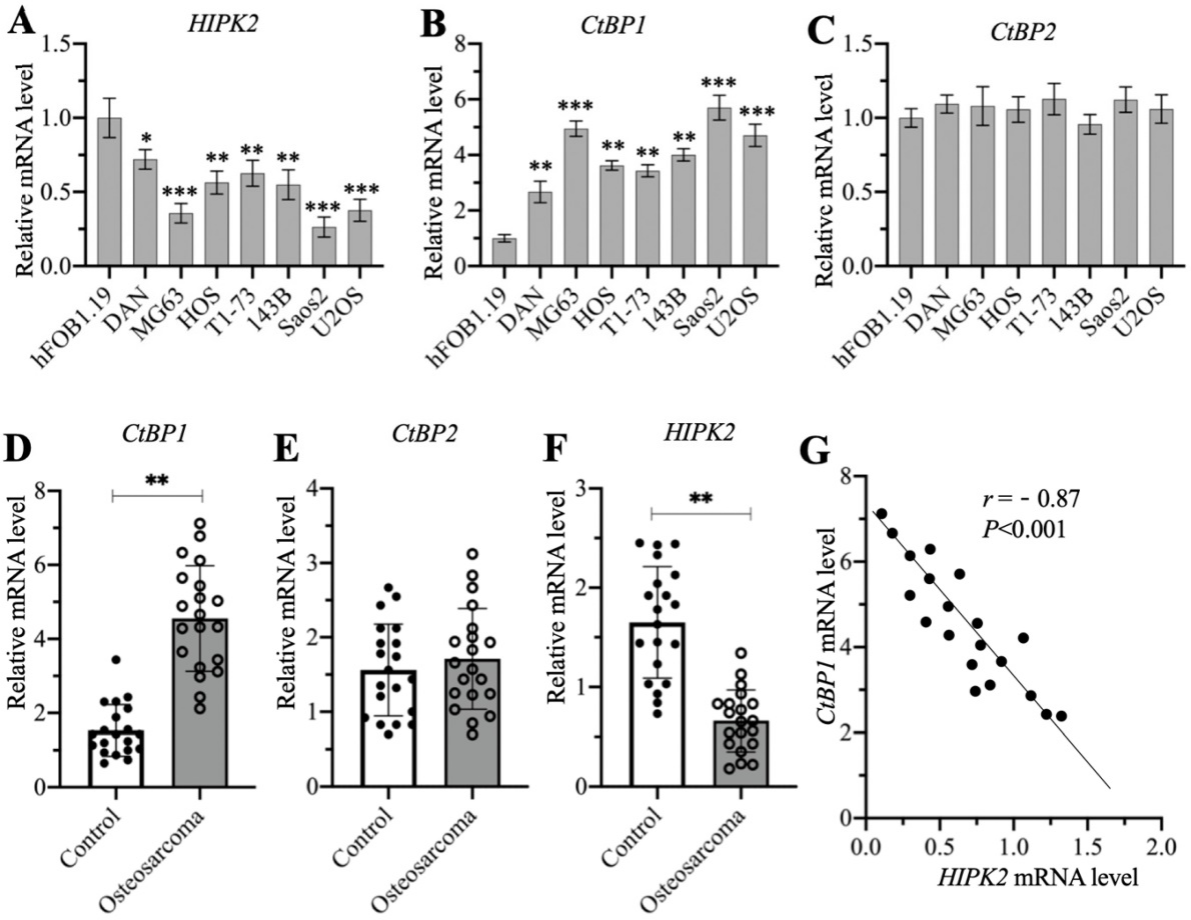
** The mRNA levels of CtBP1 and HIPK2 in osteosarcoma cells and biopsies.** (A-C) The mRNA levels of *HIPK2*, *CtBP1* and *CtBP2* in osteosarcoma cells. RNA samples from hFOB1.19, DAN, MG63, HOS, T1-73, 143B, Saos2 and U2OS cells were subjected to RT-qPCR analyses to determine the mRNA levels of *HIPK2* (A), *CtBP1* (B) and *CtBP2* (C). ^*^P <0.05, ** *P* < 0.01 and ^***^P <0.001. (D-F) The mRNA levels of *HIPK2*, *CtBP1* and *CtBP2* in biopsies. RNA samples from 20-paired cancerous and noncancerous tissues were subjected to RT-qPCR analyses to determine the mRNA levels of *CtBP1* (D), *CtBP2* (E) and *HIPK22* (F). ** *P* < 0.01. (G) The correlation assay of *CtBP1* and *HIPK2*. A Pearson correlation assay was performed using the paired relative expression levels of *CtBP1* and *HIPK2* from the same samples.

A previous publication has reported significant overexpression of *CtBP1* in osteosarcoma cells 7. Another study also showed that HIPK2 can phosphorylate CtBP1 at the Ser-422 site 19. We investigated the possible involvement of HIPK2 in the regulation of CtBP1 by examining the mRNA level of *HIPK2* in the hFOB1.19 human osteoblast cell line and in the DAN, MG63, HOS, T1-73, 143B, Saos2, and U2OS osteosarcoma cell lines. The RT-qPCR results indicated that the mRNA level of *HIPK2* showed different degrees of reduction in osteosarcoma cells, as its expression was the lowest in Saos2 cells (0.3-fold *vs* control), followed by MG63 (0.4-fold *vs* control) and U2OS (0.4-fold *vs* control), and then HOS (0.55-fold *vs* control), 143B (0.55-fold *vs* control), T1-73 (0.6-fold *vs* control) and DAN (0.7-fold *vs* control) (Figure 1A). We also examined the mRNA levels of *CtBP1* and *CtBP2* in the same cell lines and found an inverse expression pattern of *CtBP1* mRNA level in comparison to *HIPK2*. Specifically, *CtBP1* was induced approximately 6-fold in Saos2 cells, 5-fold in MG63 and U2OS cells, 3.5-4-fold in HOS, 143B and T1-73 cells, and 2.5-fold in DAN cells (Figure 1B). However, the *CtBP2* mRNA level was not significantly changed in osteosarcoma cells compared to hFOB1.19 cells (Figure 1C). We also determined whether osteosarcoma biopsies had the similar expression patterns for *CtBP1*, *CtBP2* and *HIPK2* in 20 osteosarcoma biopsies and their adjacent noncancerous tissues. The average mRNA level for* CtBP1* indicated significant overexpression in osteosarcoma biopsies compared to controls (Figure 1D). Similar to the findings in the osteosarcoma cell lines, no significant induction was evident for the *CtBP2* mRNA level in the osteosarcoma biopsies (Figure 1E). The expression of *HIPK2* was dramatically decreased in osteosarcoma biopsies (Figure 1F). Assays to determine the possible correlation between the expression of *HIPK2* and the *CtBP1* mRNA level confirmed that *CtBP1* expression was negatively correlated with *HIPK2* expression (Figure 1G). However, the expression of *CtBP2* was not correlated with *HIPK2* expression (Supplementary Figure 1).

We then determined the protein levels of CtBP1, pCtBP1, CtBP2 and HIPK2 in osteosarcoma cells and biopsies. Consistent with their mRNA levels, the CtBP1 protein level was induced approximately 4-fold in Saos2 cells, 3.3-3.5-fold in MG63 and U2OS cells, and 2-2.4-fold in HOS, 143B, T1-73 cells, and DAN cells (Supplementary Figures 2A and 2B). By contrast, the levels of phosphorylated CtBP1 and HIPK2 protein levels were decreased in osteosarcoma cells and they showed the similar patterns in the same osteosarcoma cells (Supplementary Figures 2A and 2B). The CtBP2 protein level was not changed in the osteosarcoma cells compared to hFOB1.19 cells (Supplementary Figures 2A and 2B). We also measured CtBP1, pCtBP1, CtBP2 and HIPK2 protein levels in three representative cancerous biopsies and their adjacent noncancerous tissues. The average CtBP1 protein level was increased approximately 2.7-fold, while pCtBP1 and HIPK2 levels were decreased to approximately 0.4-fold in three cancerous biopsies compared to the controls (Supplementary Figures 2C and 2D). CtBP2 protein level was also not changed in cancerous biopsies compared to controls (Supplementary Figures 2C and 2D). These results suggested that the downregulation of HIPK2 impaired the phosphorylation of CtBP1, causing CtBP1 overexpression in osteosarcoma cells and biopsies.

### Overexpression of *HIPK2* decreased only the CtBP1 protein level but not its mRNA level

**Figure 2:**
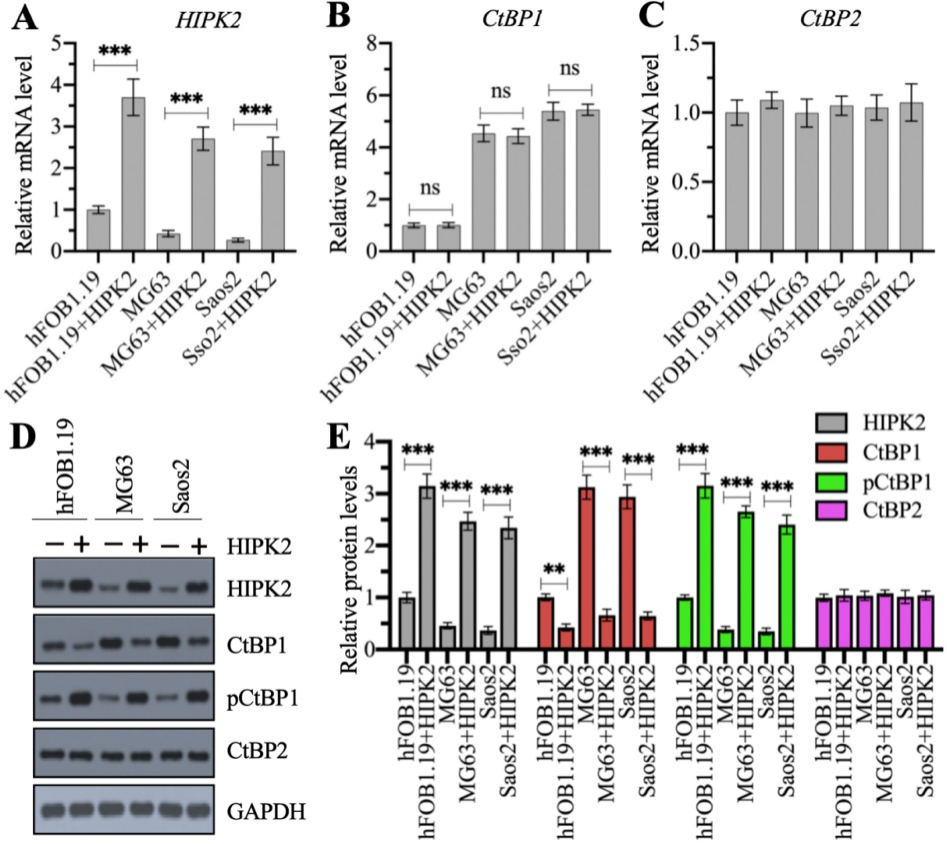
** Overexpression of HIPK2 caused the phosphorylation and degradation of CtBP1.** (A-C) The mRNA levels of *HIPK2*, *CtBP1* and *CtBP2* in HIPK2-OE cells. RNA samples from hFOB1.19+pCDNA3 (hFOB1.19), hFOB1.19+pCDNA3-HIPK2 (hFOB1.19+HIPK2), MG63+pCDNA3 (MG63), MG63+pCDNA3-HIPK2 (MG63+HIPK2), Saos2+pCDNA3 (Saos2), and Saos2+pCDNA3-HIPK2 (Saos2+HIPK2) cells were subjected to RT-qPCR analysis to determine the mRNA levels of *HIPK2* (A), *CtBP1* (B) and *CtBP2* (C). ^***^P <0.001. ns represented no significant difference. (D) The protein levels of HIPK2, CtBP1 and CtBP2 in HIPK2-OE cells. Cells used in (A) were subjected to western blotting to determine the protein levels of HIPK2, CtBP1, pCtBP1, CtBP2 and GAPDH (loading control). (E) The relative protein levels. The protein signals in (D) were quantified using Image J software and normalized to their corresponding GAPDH. ** *P* < 0.01 and ^***^P <0.001.

We examined whether the overexpression of CtBP1 mRNA and protein levels in osteosarcoma cells was directly regulated by HIPK2 by generating HIPK2-overexpression (OE) cell lines in hFOB1.19, MG63 and Saos2 backgrounds. Our RT-qPCR results showed that the successful overexpression of *HIPK2* in three background cells (Figure 2A) did not change *CtBP1* and *CtBP2* mRNA levels (Figures 2B and 2C). We then determined the protein levels of HIPK2, CtBP1, pCtBP1 and CtBP2 in these HIPK2-OE cells. As shown in Figures 2D and 2E, we found that overexpression of *HIPK2* caused the decrease of CtBP1 protein level but increased the pCtBP1 level. However, the CtBP2 protein level was not changed in these cells (Figures 2D and 2E). These results suggested that *HIPK2* overexpression only affected CtBP1 protein level but not its mRNA level.

### Overexpression of *HIPK2* increased the sensitivity of osteosarcoma cells to chemotherapeutic drugs

**Figure 3:**
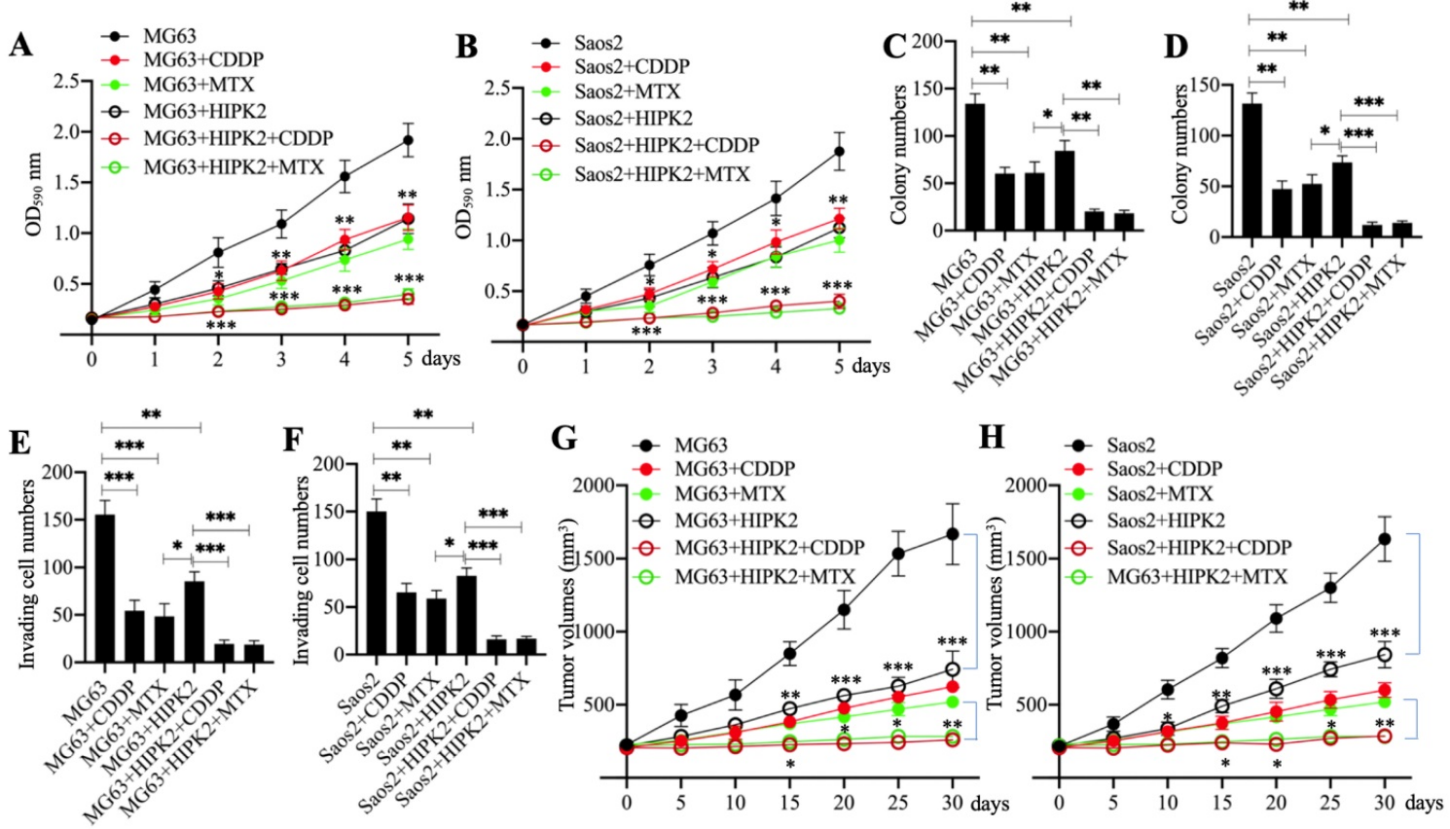
** Overexpression of HIPK2 inhibited osteosarcoma cell growth *in vitro* and *in vivo.***(A and B) MTT assay results. The MG63 (MG63+pCDNA3), MG63+HIPK2, and Saos2 (Saos2+pCDNA3), Saos2+HIPK2 cells were seeded into DMEM containing DMSO, 25 µM CDDP or 25 µM MTX and cultured at 37°C. Cell viability was measured with an MTT kit daily. * *P* < 0.05, ** *P* < 0.01 and *** *P* < 0.001. (C and D) Colony numbers: the same cells as indicated in (A) and (B) were seeded into six-well plates and grown in DMEM containing DMSO, 25 µM CDDP or 25 µM MTX for 14 days with medium renewal every three days. Colony numbers were counted manually. * *P* < 0.05, ** *P* < 0.01 and *** *P* < 0.001. (E and F) The invading cell numbers. Cells used in (A) were used for Boyden chamber assay and the invading cells were counted manually. * *P* < 0.05, ** *P* < 0.01 and *** *P* < 0.001. (G and H) Tumor volumes *in vivo*. Cells used in (A) were injected into nude mice (n=30 for each cell line). Mice with similar tumor volumes (approximately 200 mm^3^) in each group were divided into three subgroups and injected with PBS, CDDP or MTX at 5-day intervals. Tumor volumes were measured at 5-day intervals for 30 days. ^*^P <0.05, ** *P* < 0.01 and ^***^P <0.001.

HIPK2 functions as a tumor suppressor in osteosarcoma cells; therefore, we speculated that its overexpression should inhibit osteosarcoma cell growth. To confirm this speculation, we generated the Control-OE and HIPK2-OE cells in MG63 and Saos2 backgrounds and then measured cell proliferation, colony formation and cell invasion in response to treatments with the chemotherapeutic drugs CDDP and MTX. The MTT assay results showed that overexpression of *HIPK2* in both MG63 and Saos2 backgrounds significantly decreased cell proliferation in comparison to Control-OE cells (Figures 3A and 3B). The viability of HIPK2-OE cells treated with either CDDP or MTX was also significantly decreased in comparison to the untreated HIPK2-OE cells and when compared to the CDDP-treated or MTX-treated Control-OE cells (Figures 3A and 3B). The numbers of colonies and invading cells were also markedly decreased in HIPK2-OE cells compared to Control-OE cells in the conditions even without chemotherapeutic drug treatments (Figures 3C-3F and Supplementary Figures 3A and 3B). Treatments with CDDP and MTX further reduced the numbers of colonies and invading cells in the HIPK2-OE cells compared to the untreated HIPK2-OE cells and the CDDP-treated or MTX-treated Control-OE cells (Figures 3C-3F and Supplementary Figures 3A and 3B).

We also determined the *in vivo* effect of *HIPK2* overexpression by injecting Control-OE and HIPK2-OE cells into nude mice to generate tumors. Mice harboring similar tumor volumes were divided into three groups and injected with PBS, CDDP, or MTX. Tumor volume monitoring showed that overexpression of *HIPK2* significantly inhibited tumor growth (Figures 3G and 3H). The administration of CDDP and MTX also significantly decreased the tumor volumes in mice injected with HIPK2-OE cells compared with mice injected with Control-OE cells (Figures 3G and 3H). These results suggested that overexpression of *HIPK2* could inhibit osteosarcoma cell growth both *in vitro* and *in vivo*, and could also increase the chemosensitivity to CDDP and MTX.

### Overexpression of *HIPK2* induced the expression of proapoptotic genes

The observation that overexpression of *HIPK2* could inhibit osteosarcoma cell growth *in vitro* and *in vivo* prompted us to determine the gene expression changes following *HIPK2* overexpression. For this purpose, we conducted a microarray assay using RNA samples from Control-OE and HIPK2-OE cells in both MG63 and Saos2 backgrounds. A total of 28 differentially expressed genes were found in both MG63 and Saos2 backgrounds of HIPK2-OE cells (Figure 3A and Supplementary Table 3). Among these upregulated genes, we found six proapoptotic genes: *BIM*, *BAX*, *BIK*, *NOXA*, *PUMA*, and *PERP* (P53 apoptosis effector related to PMP22) (Figure 3A and Supplementary Table 3). These proapoptotic genes are all the known targets of CtBP1, suggesting that *HIPK2* overexpression inhibited tumor growth mainly by inducing the expression of proapoptotic genes.

**Figure 4:**
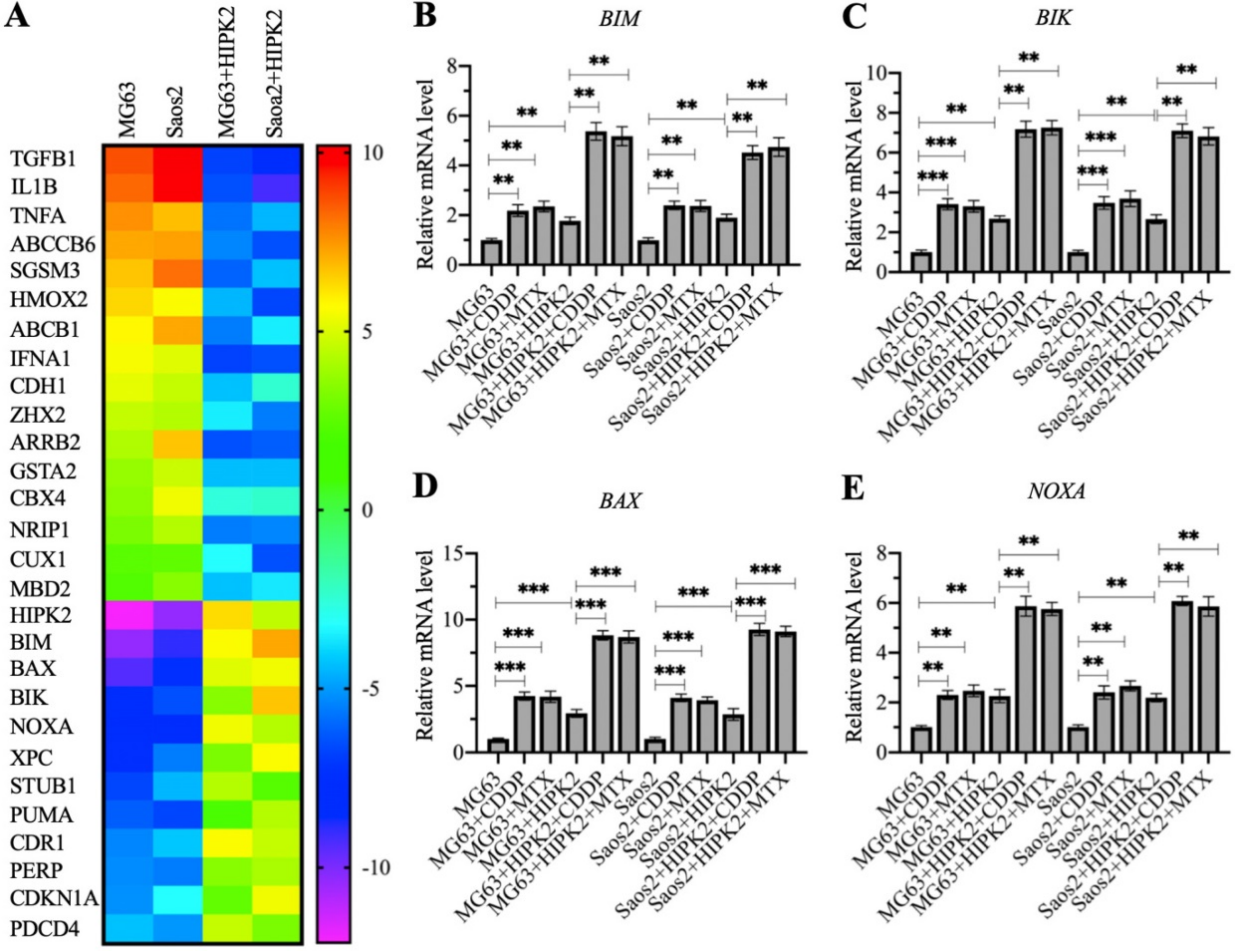
** Identification of the differentially expressed genes in HIPK2-OE cells by microarray assay.** (A) Microarray results. RNA samples from MG63, Saos2, MG63+HIPK2, Saos2+HIPK2 cells were subjected to microarray analysis. (B-E) Verification of gene expression. RNA samples from MG63, MG63+CDDP, MG63+MTX, MG63+HIPK2, MG63+HIPK2+CDDP, MG63+HIPK2+MTX, Saos2, Saos2+CDDP, Saos2+MTX, Saos2+HIPK2, Saos2+HIPK2+CDDP, and Saos2+HIPK2+MTX cells were subjected to RT-qPCR analyses to detect mRNA levels of *BIM* (B),* BIK* (C), *BAX* (D), and *NOXA* (E). *P* <0.05, ** *P* < 0.01 and ^***^P <0.001.

To determine the accuracy of microarray results and detect the expression levels of proapoptotic genes in CDDP-treated and MTX-treated cells, we performed RT-qPCR analyses to detect four representative proapoptotic genes, including *BIM*, *BIK*, *BAX* and *PUMA*, and two non-apoptotic genes, including *TGFB1* (Transforming growth factor beta 1) and *HMOX2* (Heme oxygenase 2). Consistent with the microarray results, we found that *HIPK2* overexpression significantly induced the expression of *BIM*, *BIK*, *BAX* and *PUMA*, but decreased the expression of *TGFB1* and *HMOX2* (Figures 4B-4E and Supplementary Figure 4). Comparison of the expression levels in CDDP-treated, MTX-treated, and untreated cells, we found that the CDDP and MTX treatments significantly induced the expression of the proapoptotic genes but not of *TGFB1* and *HMOX2* (Figures 4B-4E and Supplementary Figure 4). These results, together with the *in vitro* and *in vivo* tumor growth inhibition results, suggested that overexpression of *HIPK2* and the use of chemotherapeutic drugs could both increase the expression of proapoptotic genes, thereby suppressing tumor cell growth.

### Overexpression of *HIPK2* caused the degradation of CtBP1 and induced apoptosis

**Figure 5:**
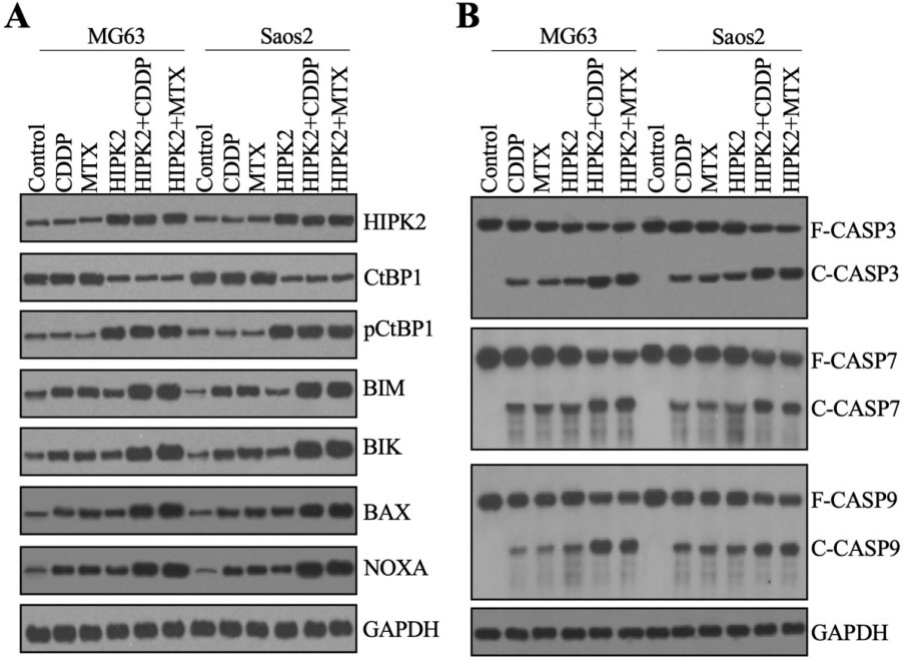
** Overexpression of HIPK2 induced apoptosis.** (A) The protein levels of proapoptotic proteins. Total cell extracts from MG63, MG63+CDDP, MG63+MTX, MG63+HIPK2, MG63+HIPK2+CDDP, MG63+HIPK2+MTX, Saos2, Saos2+CDDP, Saos2+MTX, Saos2+HIPK2, Saos2+HIPK2+CDDP, and Saos2+HIPK2+MTX cells were subjected to western blotting to examine protein levels of HIPK2, CtBP1, pCtBP1, BIM, BIK, VAX, NOXA, and GAPDH (loading control). (B) The protein levels of caspases. The same protein samples used in (A) were subjected to western blotting to examine the protein levels of CSAP3, CASP7, CASP9, and GAPDH (loading control).

The discovery that overexpression of *HIPK2* resulted in the upregulation of CtBP1 targets promoted us to examine the protein levels of CtBP1 and proapoptotic proteins. We used the total protein extracts from CDDP-treated and MTX-treated and untreated Control-OE and HIPK2-OE cells from both MG63 and Saos2 backgrounds to examine the HIPK2 protein level. We found an approximately 3-fold induction of the HIPK2 protein level in HIPK2-OE cells compared to Control-OE cells (Figure 5A and Supplementary Figure 5). Neither CDDP nor MTX treatment changed the HIPK2 protein level (Figure 5A and Supplementary Figure 5). Subsequent examination of the CtBP1 and pCtBP1 protein levels revealed an inverse pattern (Figure 5A and Supplementary Figure 5), as CtBP1 levels were significantly decreased while pCtBP1 levels were markedly increased in HIPK2-OE cells (Figure 5A and Supplementary Figure 5). Similar to the effects observed for HIPK2, neither CDDP nor MTX treatment changed the CtBP1 or pCtBP1 protein levels (Figure 5A and Supplementary Figure 5). Using the same protein samples, we also observed an induction of BIM, BIK, BAX and NOXA in HIPK2-OE cells and an enhancement of this induction by CDDP and MTX treatments (Figure 5A and Supplementary Figure 5).

The induction of proapoptotic proteins implied the possible activation of intrinsic apoptosis signaling. We verified this possibility by also determining the protein levels of the apoptotic markers CASP3, CASP7 and CASP9. The immunoblot results indicated that all these three caspases were activated in HIPK2-OE cells, even without chemotherapeutic drug treatments (Figure 5B and Supplementary Figure 6). Both CDDP and MTX could activate CASP3, CASP7 and CASP9 in Control-OE cells, and both caused a much high induction of caspases in the HIPK2-OE cells (Figure 5B and Supplementary Figure 6). These results suggested that caspases were in different activation states in Control-OE and HIPK2-OE cells treated with or without chemotherapeutic drugs, and these different states could explain the *in vitro* and *in vivo* phenotypes for the inhibition of tumor cell growth.

### The CtBP1-p300-FOXO3a complex bound to the promoters of proapoptotic genes to control their expression

**Figure 6:**
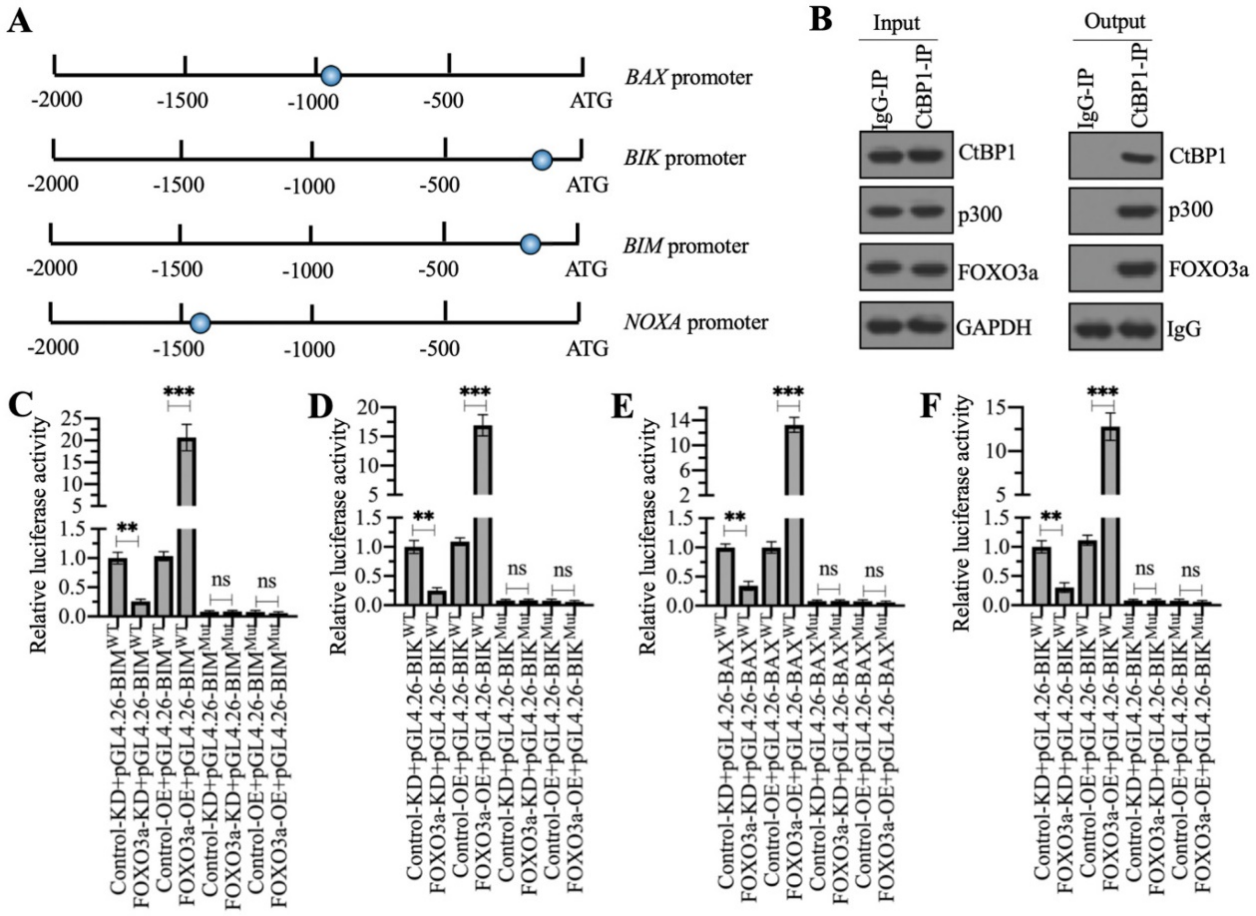
**The CtBP1-p300-FOXO3a complex bound to the promoters of proapoptotic genes.** (A) Schematic diagrams of FOXO3a binding sites on the promoters of proapoptotic genes. A 1500-bp length of each proapoptotic gene promoters was used to identify the FOXO3a-binding sites and the binding sites were shown as blue circles. (B) The assembly of CtBP1-p300-FOXO3a complex. Total cell extract from MG63 cells was applied to immunoprecipitation assays using anti-CtBP1 and IgG. The input and output proteins were subjected to western blotting assays to determine the protein levels of CtBP1, p300 and FOXO3a. (C-F) Luciferase assays. The Control-KD, FOXO3a-KD, Control-OE and FOXO3a-OE cells were co-transfected pGL4.26-pBIM^WT^ (or pGL4.26-pBIM^Mut^) + Renilla (C), pGL4.26-pBIK^WT^ (or pGL4.26-pBIK^Mut^) + Renilla (D), pGL4.26-pBAX^WT^ (or pGL4.26-pBAX^Mut^) + Renilla (E), and pGL4.26-pNOXA^WT^ (or pGL4.26-pNOXA^Mut^) + Renilla (F), respectively. After culturing for 24 h, the cells were subjected to luciferase assays. ** *P* < 0.01 and ^***^P <0.001. ns represented no significant difference.

As mentioned earlier, a previous study has indicated that CtBP1 couples with p300 and FOXO3a to regulate the expression of *BAX* and *BIM* in osteosarcoma cells 7. We validated the conservation of this regulatory mechanism in the regulation of other proapoptotic genes by analyzing the sequences of the *BAX*, *BIK*, *BIM* and *NOXA* promoters to confirm the presence of FOXO3a binding sites. Using the FOXO3a consensus site (A/G)TAAA(T/C)A to screen the promoters of proapoptotic genes, we found all these four gene promoters contained a FOXO3a binding site (Figure 6A). We then examined whether the CtBP1-p300-FOXO3a complex was responsible for the regulation of these proapoptotic genes by immunoprecipitation assays in the MG63 cell lysate using anti-CtBP1- and IgG-coupled protein A beads. The input and output proteins were subjected to western blotting to examine the protein levels of CtBP1, p300, and FOXO3a, respectively. Our results showed that CtBP1 but not IgG pulled down both p300 and FOXO3a (Figure 6B).

We then validated that the FOXO3a-coupled complex mediated the expression of proapoptotic genes through the consensus sites shown in Figure 6A by creating luciferase vectors containing the WT or mutated promoters of the proapoptotic genes and transfecting these plasmids into Control-KD, FOXO3a-KD, Control-OE and FOXO3a-OE cells. Subsequent luciferase assays showed significantly lower luciferase activities in FOXO3a-KD cells expressing the WT promoters of* BAX*, *BIK*, *BIM,* and *NOXA* than in the Control-KD cells (Figures 6C-6F). Conversely, the luciferase activities were much higher in FOXO3a-OE cells expressing the WT promoters of* BAX*, *BIK*, *BIM* and *NOXA* than in the Control-OE cell (Figures 6C-6F). The luciferase activities in all cells transfected with the mutated promoters were similar and were maintained at the basal levels (Figures 6C-6F). These results suggested that FOXO3a bound to the promoters of proapoptotic genes through the consensus sites.

### Changing the expression of *HIPK2* and *CtBP1* caused a reversal of the effects on proapoptotic gene levels

**Figure 7:**
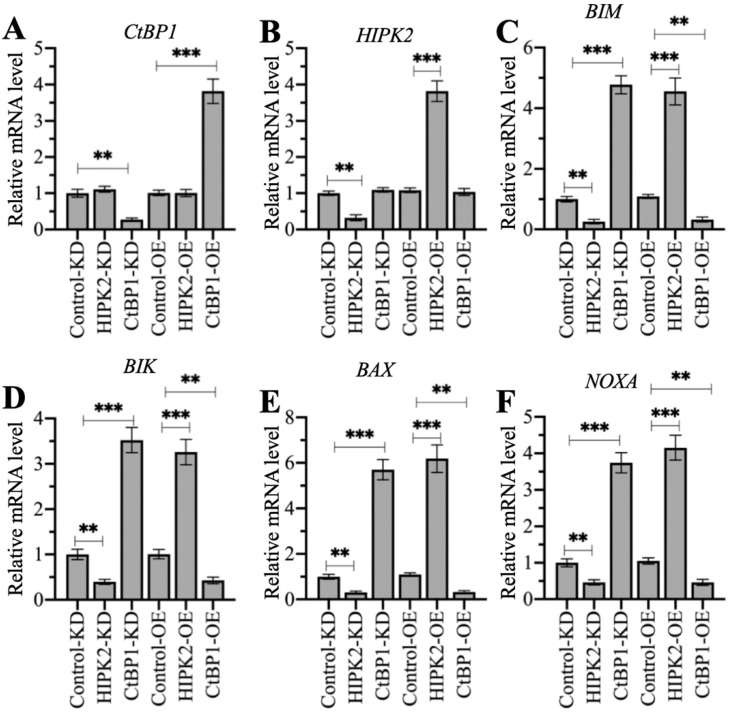
** The different effects of depicting *HIPK2* and *CtBP1* expression levels on proapoptotic gene expression.** RNA samples from Control-KD, HIPK2-KD, CtBP1-KD, Control-OE, HIPK2-OE, and CtBP1-OE cells were subjected to RT-qPCR analyses to examine the mRNA levels of *CtBP1* (A), *HIPK2* (B), *BIM* (C), *BIK* (D), *BAX* (E), and *NOXA* (F). ** *P* < 0.01 and ^***^P <0.001.

HIPK2 phosphorylates CtBP1 and cause its degradation, thereby inducing the expression of proapoptotic genes in osteosarcoma cells. Therefore, we speculated that knockdown of *HIPK2* in hFOB1.19 cells should reduce proapoptotic gene expression as that in osteosarcoma cells, while overexpression of *HIPK2* in hFOB1.19 cells should induce proapoptotic gene expression. The knockdown and the overexpression of *CtBP1* in hFOB1.19 cells should have inverse effects on proapoptotic gene expression. We verified this speculation by generating Control-KD, HIPK2-KD, CtBP1-KD, Control-OE, HIPK2-OE, and CtBP1-OE cells (Supplementary Figure 7), and then measuring the mRNA levels of *HIPK2*, *CtBP1*, and four proapoptotic genes. Neither knockdown nor overexpression of *HIPK2* changed the *CtBP1* mRNA level, but they did cause the decrease or increase, respectively, of proapoptotic genes (Figures 7A-7F). By contrast, knockdown or overexpression of *CtBP1* resulted in the upregulation or downregulation, respectively, of proapoptotic genes (Figures 7C-7F). However, neither knockdown nor overexpression of *CtBP1* could not change the HIPK2 mRNA level (Figure 7B). These results suggest that HIPK2 does not mediate CtBP1 at the transcriptional level, and that its knockdown or overexpression probably changes the phosphorylation status of CtBP1, thereby affecting the subsequent expression of proapoptotic genes.

## Discussion

**Figure 8:**
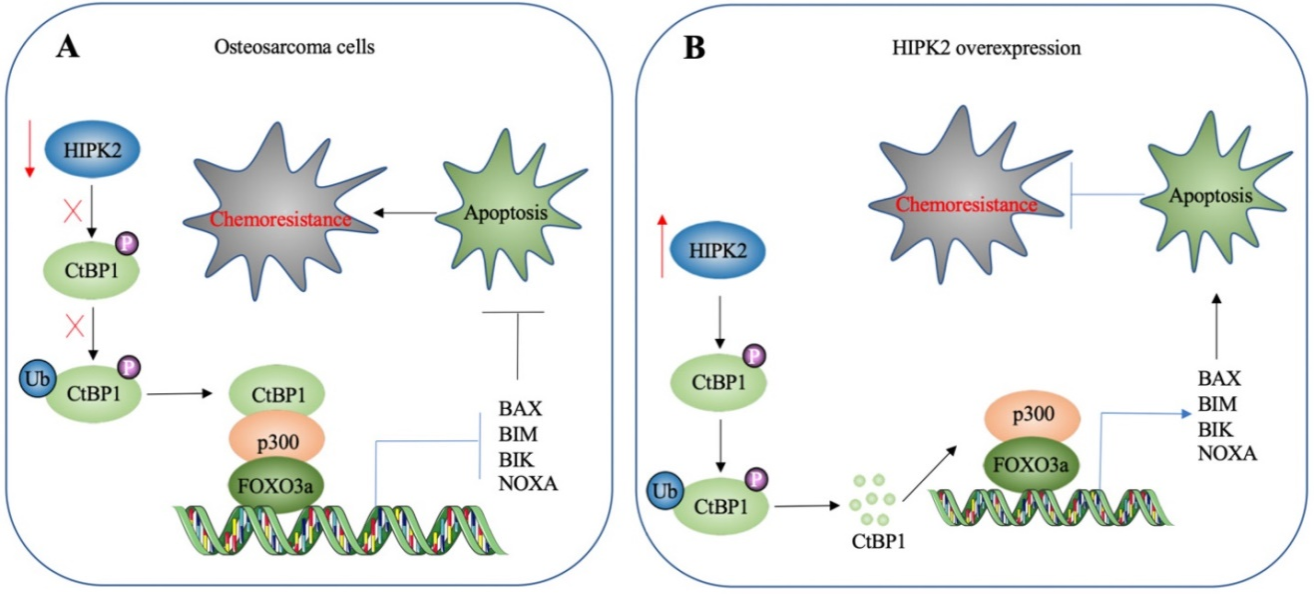
** A schematic diagram HIPK2-mediated activation of apoptosis in osteosarcoma cells.** (A) A schematic diagram of HIPK2 downregulation in osteosarcoma cells. The downregulation of HIPK2 results in failure to phosphorylate CtBP1, thereby impairing the ubiquitination and degradation of CtBP1. The accumulated CtBP1 assembles as a complex with p300 and FOXO3a, and the complex then docks onto the promoters of proapoptotic genes to repress their expression. This results in the inhibition of apoptosis, thereby increasing the chemoresistance of the cells. (B) A schematic diagram of HIPK2-overexpression in osteosarcoma cells. The overexpression of HIPK2 causes CtBP1 phosphorylation, thereby promoting the ubiquitination and degradation of CtBP1. The decreased CtBP1 levels prevent the formation of the complex with p300 and FOXO3a, resulting in an increase in proapoptotic gene expression. The induction of proapoptotic gene expression then promotes apoptosis and decreases chemoresistance.

HIPK2 is a known tumor suppressor that can phosphorylate CtBP1 at the Ser-422 site 19. However, whether HIPK2 functions as a tumor suppressor that phosphorylates CtBP1 to promote the tumorigenesis of osteosarcoma is not known. The findings presented here revealed a significant downregulation of HIPK2 in both osteosarcoma cell lines and biopsies, as well as an impairment of CtBP1 phosphorylation and a consequent accumulation of CtBP1. CtBP1, in turn, was assembled into a complex with p300 and FOXO3a, and this transcriptional complex then bound to the promoters of proapoptotic genes to repress their expression. This downregulation of proapoptotic genes inhibited apoptosis, thereby leading to chemoresistance (Figure 8A). By contrast, overexpression of *HIPK2* increased the phosphorylation of CtBP1 and promoted its degradation through the proteasomal pathway. This degradation of CtBP1 prevented its repression of proapoptotic genes, thereby allowing apoptosis and resulting in increased chemosensitivity (Figure 8B).

HIPK2 can modulate different biological processes, including apoptosis, cell proliferation and differentiation, metastasis and the EMT 22-26. However, a role for HIPK2 in these processes has not been established for osteosarcoma. Similar to many other cancer types, the osteosarcoma cells and biopsies showed significantly decreased expression of HIPK2, whereas its overexpression suppressed cell viability and increased chemosensitivity following CDDP and MTX treatments. The current study has mainly focused on how HIPK2 overexpression results in apoptosis. However, apart from the proapoptotic genes, we also found the decrease in expression of *CDH1*, an EMT marker, in HIPK2-OE cells (Figure 4A and Supplementary Table 3). In agreement with this finding, several studies have also shown that forced expression of HIPK2 can inhibit the EMT process. We also found downregulation of the expression of TGFB1, a critical molecule involved in cell invasion and tumor metastasis, in HIPK2-OE cells (Figure 4A and Supplementary Table 3). These results suggest that overexpression of HIPK2 may affect the expression of genes involved in many different biological processes, with the CtBP1-p300-FOXO3a-mediated apoptosis signaling being one of these processes. Both *CDH1* and *TGFB1* are the targets of CtBP1 in other types of tumor cells; however, we cannot conclude that they are controlled by CtBP1 in the tumorigenesis of osteosarcoma because many transcription factors can regulate the expression of *CDH1* and *TGFB1*. More efforts are needed to study the influences of *HIPK2* overexpression, especially its effects on the EMT and metastasis. However, the promising result observed here that HIPK2 overexpression can inhibit osteosarcoma cell growth *in vitro* and *in vivo* suggests that HIPK2 may be a therapeutic target for the treatment of osteosarcoma.

CtBP1 is a well-known transcription regulator of genes involved in tumorigenesis, progression and metastasis 12. However, the mechanism underlying its overexpression is still obscure. Apart from its regulation by HIPK2-mediated phosphorylation at the post-transcriptional level, CtBP1 can be regulated by a microRNA called miR-485-3p 27. One interesting result in Figure 2 indicates that knockdown or overexpression of *HIPK2* did not change *CtBP1* mRNA level. However, we identified overexpression of *CtBP1* mRNA level in osteosarcoma cells and biopsies (Figure 1). These results suggest that CtBP1 expression is mediated at both transcriptional and translational levels. Although we identified a HIPK2-mediated phosphorylation mechanism in the present study, the mechanism for transcriptional regulation of *CtBP1* in osteosarcoma cells is not known.

In summary, our study demonstrates that overexpression of *HIPK2* enhances the phosphorylation of CtBP1 and promotes its degradation by the proteasomes, thereby disrupting the formation of the CtBP1-p300-FOXO3a complex that binds to promoters of proapoptotic genes. This disruption allows the expression of these proapoptotic genes, resulting in apoptosis and an increased chemosensitivity to chemotherapeutic drugs.

## Supplementary Material

Supplementary figures and tables.
