# Supplementary material for: Overexpression of HIPK2 removes the transrepression of proapoptotic genes mediated by the CtBP1-p300-FOXO3a complex and increases the chemosensitivity in osteosarcoma cells
Source: J Cancer. 2021 Jan 21;12(6):1826–37. doi: 10.7150/jca.52115 (PMC7890331; doi:10.7150/jca.52115)
Supplement: Supplementary file 1 — Supplementary figures and tables. [file jcav12p1826s1.pdf]

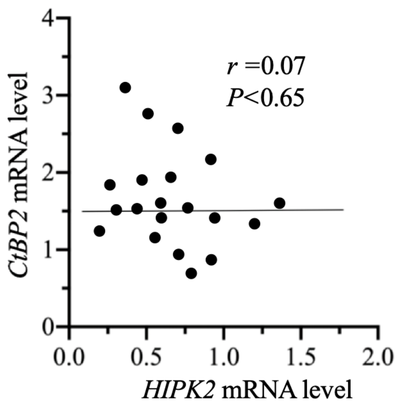

Supplementary Figure 1. ***CtBP2*** **mRNA level was not correlated with** ***HIPK2***

A Pearson correlation assay was performed using the paired relative expression levels of *CtBP2* and *HIPK2* from the same samples.

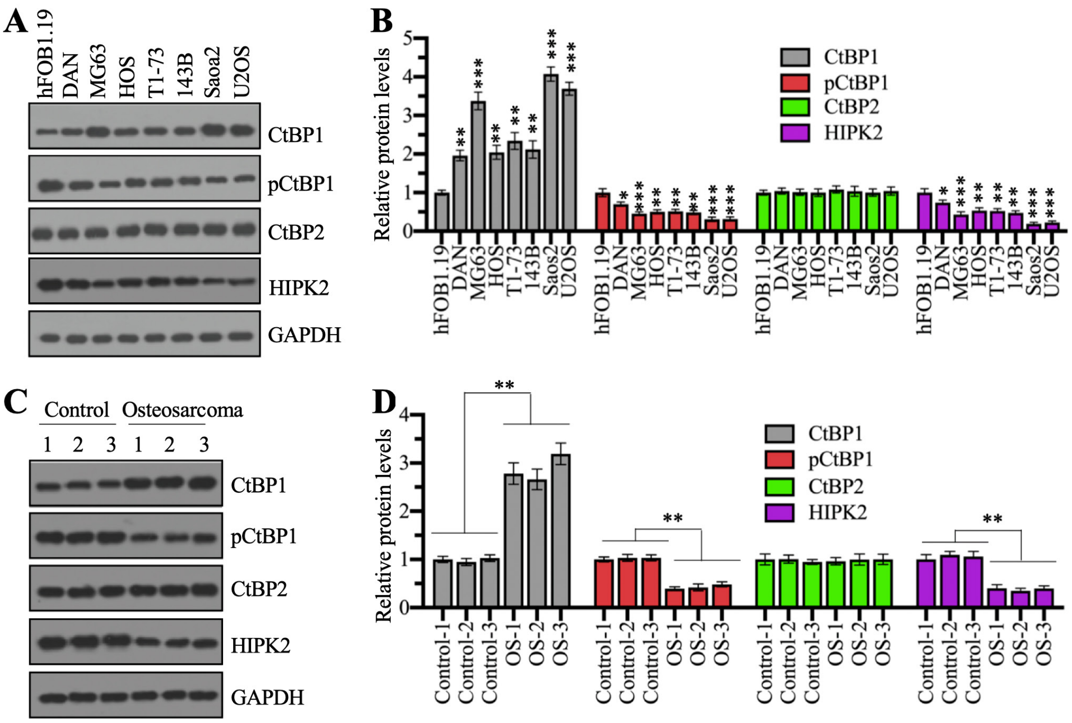

Supplementary Figure 2. The protein levels of CtBP1 and HIPK2 in osteosarcoma cells and biopsies

(A) The protein levels of CtBP1 and HIPK2 in osteosarcoma cells. Total proteins from hFOB1.19, DAN, MG63, HOS, T1-73, 143B, Saos2 and U2OS cells were subjected to western blotting to examine the protein levels of CtBP1, pCtBP1, CtBP2, HIPK2 and GAPDH (loading control). **(B)** The relative protein levels of CtBP1 and HIPK2 in osteosarcoma cells. The protein signals in (A) were quantified using Image J software and normalized to their corresponding GAPDH. $^{*}P$ <0.05, ** P < 0.01 and $^{***}P$ <0.001. **(C)** The protein levels of CtBP1 and HIPK2 in osteosarcoma biopsies. Total proteins from three representative osteosarcoma biopsies and their adjacent noncancerous tissues were subjected to western blotting to examine the protein levels of CtBP1, pCtBP1, CtBP2, HIPK2 and GAPDH (loading control). **(D)** The relative

protein levels of CtBP1 and HIPK2 in osteosarcoma biopsies. The protein signals in (C) were quantified using Image J software and normalized to their corresponding GAPDH. $^{*}P$ <0.05, ** P < 0.01 and $^{***}P$ <0.001.

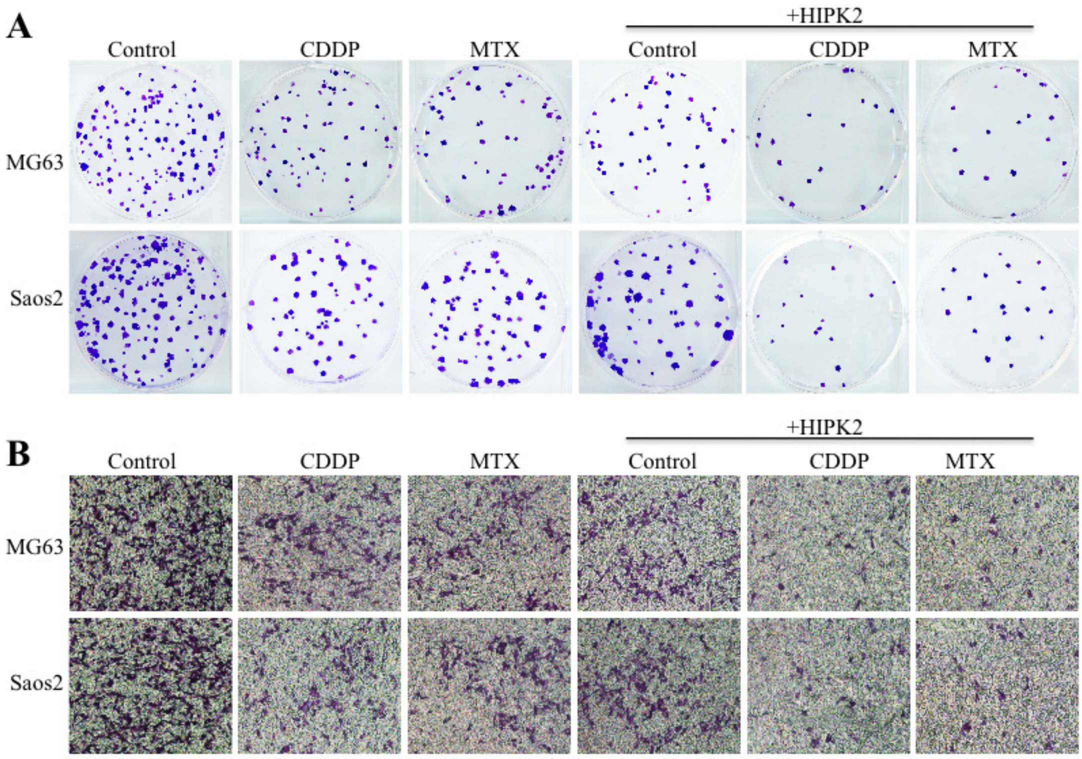

Supplementary Figure 3. Overexpression of HIPK2 decreased the colony numbers and invading cell numbers

(A) Colony formation results. The MG63 (MG63+pCDNA3), MG63+HIPK2, Saos2 (Saos2+pCDNA3), Saos2+HIPK2 cells were seeded into six-well plates and grown in DMEM containing DMSO, 25 μM CDDP or 25 μM MTX for 14 days with medium renewal every three days. Colonies were stained with 0.1% crystal violet and then photographed. **(B)** Cell invasion results. The MG63 (MG63+pCDNA3), MG63+HIPK2, Saos2 (Saos2+pCDNA3), Saos2+HIPK2 cells were subjected to Boyden chamber assays. The invaded cells were stained with 0.1% crystal violet and then photographed.

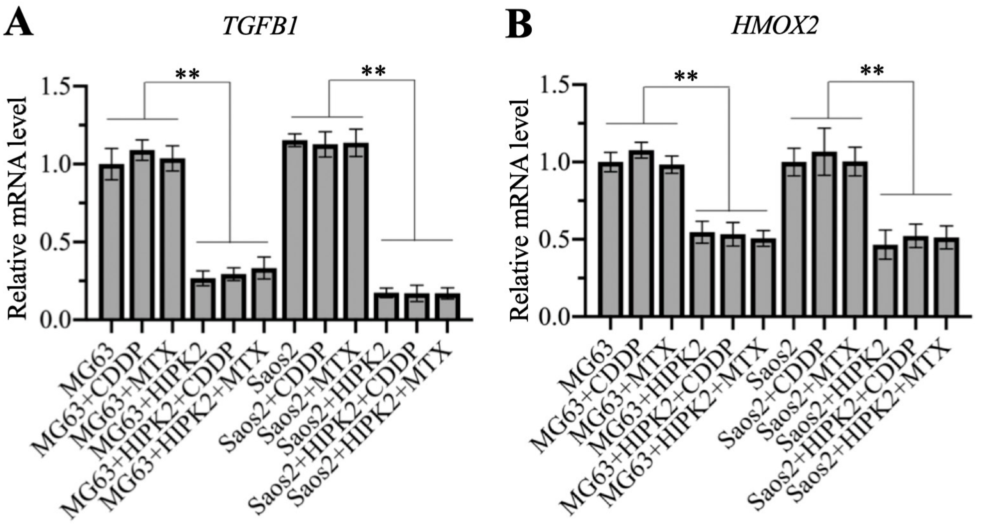

Supplementary Figure 4. The mRNA levels of *TGFB1* and *HMOX2* in HIPK2-OE cells treated with or without chemotherapeutic drugs

RNA samples from MG63, MG63+CDDP, MG63+MTX, MG63+HIPK2, MG63+HIPK2+CDDP, MG63+HIPK2+MTX, Saos2, Saos2+CDDP, Saos2+MTX, Saos2+HIPK2, Saos2+HIPK2+CDDP, and Saos2+HIPK2+MTX cells were subjected to RT-qPCR analyses to detect mRNA levels of *TGFB1* **(A)**, and *HMOX2* **(B)**. $P<0.05$, ** $P < 0.01$ and $^{***}P<0.001$.

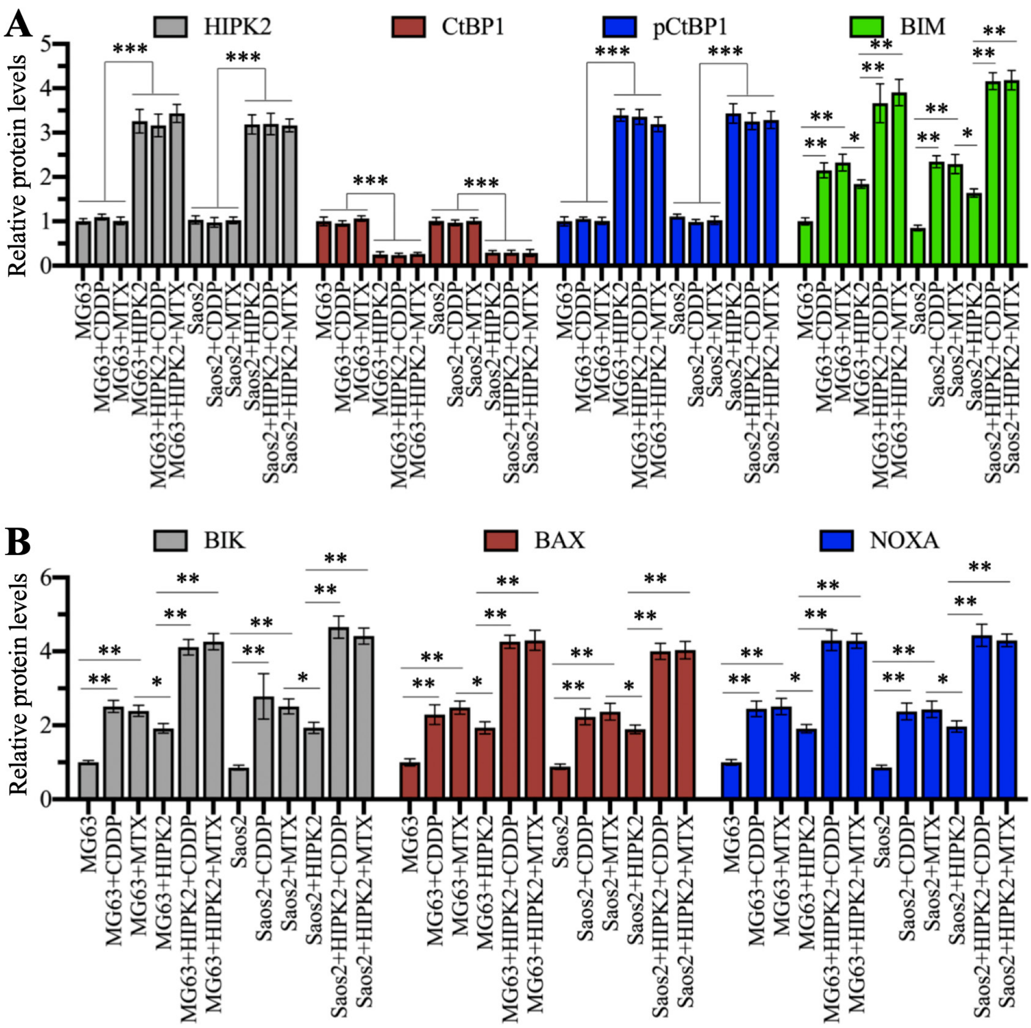

Supplementary Figure 5. The relative protein levels of proapoptotic proteins and caspases

(A) The relative protein levels of HIPK2, CtBP1, pCtBP1 and BIM. The protein signals of HIPK2, CtBP1, pCtBP1 and BIM in Figure 5A were quantified using Image J software and normalized to their corresponding GAPDH. $^{*}P$ <0.05, ** P < 0.01 and $^{***}P$ <0.001. **(B)** The relative protein levels of BIK, BAX and NOXA. The protein signals of BIK, BAX and NOXA in Figure 5A were quantified using Image J

software and normalized to their corresponding GAPDH. $^{*}P$ <0.05, ** P < 0.01 and $^{***}P$ <0.001.

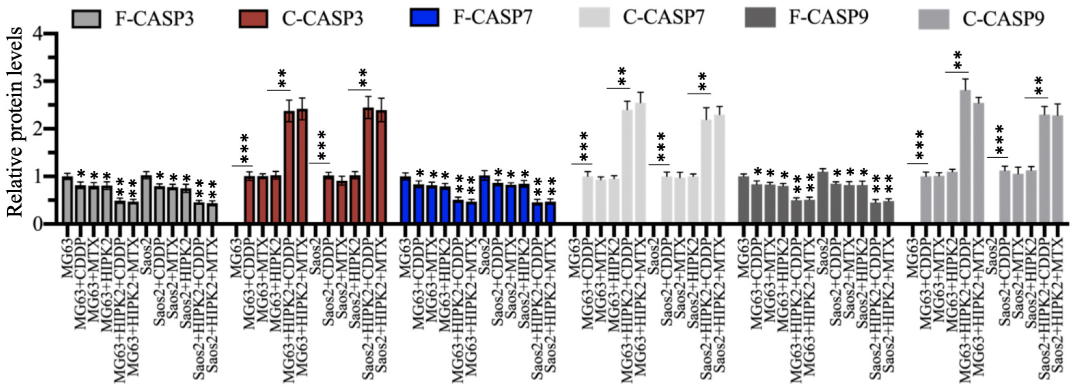

Supplementary Figure 6. The relative protein levels of caspases

The protein signals in Figure 5B were quantified using Image J software and normalized to their corresponding GAPDH. *P <0.05, ** P < 0.01 and ***P <0.001.

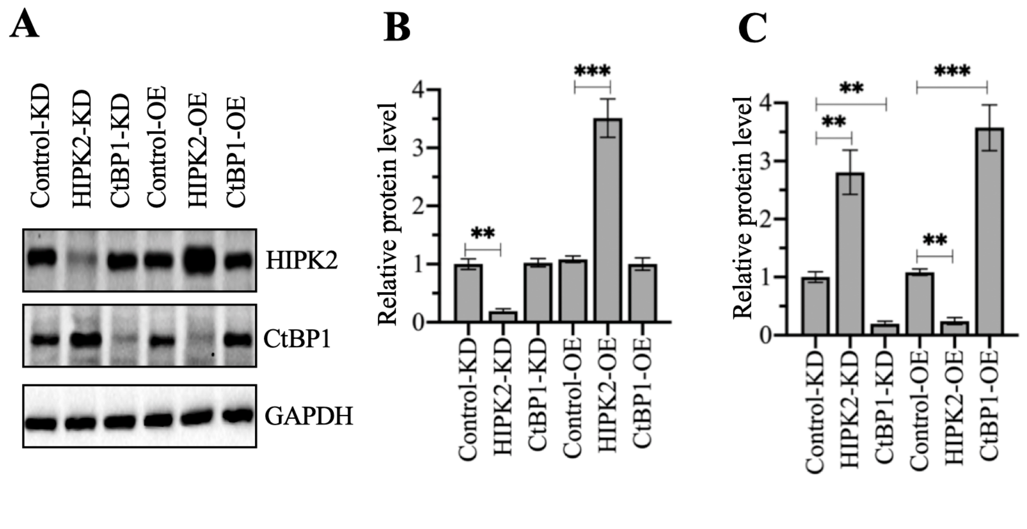

Supplementary Figure 7. Protein levels of HIPK2 and CtBP1 in HIPK2-KD, HIPK2-OE, CtBP1-KD, and CtBP1-OE cells

(A) Protein levels of HIPK2 and CtBP1. Total cell extracts from Control-KD, HIPK2-KD, CtBP1-KD, Control-OE, HIPK2-OE, and CtBP1-OE cells were used for western blotting to determine the protein levels of HIPK2, CtBP1, and GAPDH. **(B and C)** The relative protein levels of HIPK2 and CtBP1. The protein signals shown in (A) were quantified and normalized to GAPDH. **(B)** The relative protein level of HIPK2. **(C)** The relative protein level of CtBP1. ** $P < 0.01$ and $^{***}P < 0.001$.

Supplementary Table-1. Primers used for vector constructions

Vector	Forward (5'-3')	Reverse (5'-3')
pCDNA3-CtBP1	CGGGATCCATGGGCAGCTCGCACTTG	CCGCTCGAGCTACAACTGGTCACTGGCGTGGT
pCDNA3-HIPK2	CGGGATCCATGGCCCCCGTGTACGAAGGTAT	CCGCTCGAGTTATATGTAAGGGTACTGGT
pGL4.26-pBIM^{WT}	CGAGCTCGTCTCGGGGGACGCATGAACCC	CCGCTCGAGGAAACCTGCGCGGCCCTGCAGC
pGL4.26-pBIM^{Mut}	AGTTACTCCGCAGGGAGCGCCAG	CTGGCGCTCCCTGCGGAGTAACT
pGL4.26-pBIK^{WT}	CGAGCTCAAACACCTGACCTCAGGTGATC	CCGCTCGAGGCGGCCCGGCTGCCGGCGC
pGL4.26-pBIK^{Mut}	AAGACAGAACAGGGAGAGCTTTGC	GCAAAGCTCTCCCTGTTCTGTCTT
pGL4.26-pBAX^{WT}	CGAGCTCGGTGGATGAAAAAAACCAACATGA	CCGCTCGAGGCCCGGGTCACGTGAGAGCC
pGL4.26-pBAX^{Mut}	TATACCCATCAGGGAGCCATTCAG	CTGAATGGCTCCCTGATGGGTATA
pGL4.26-pNOXA^{WT}	CGAGCTCCCTTATGTATTAGGTAAGTC	CCGCTCGAGGGTGACGTAGGGAAACTAGAC
pGL4.26-pNOXA^{Mut}	CTCGTTTTTGCAGGGAGTCCACA	TGTGGACTCCCTGCAAAAACGAG

Supplementary Table-2. Primers used for RT-qPCR analyses

Gene	Forward	Reverse
HIPK2	CCACGTGAACTCAAGAGAACATG	GGTTATCTGTTCTCAATGAC
CtBP1	ACTGTGTCAACAAGGACCATC	TACCTTCCACAGCAGCTGGGA
CtBP2	ATGGCAATCTGGGAGCAGC	TATTCCAGATTCTGGGCAGTG
BIM	ACCACCCACGAATGGTTATC	CGGTGCTGGGTCTTGTTGGT
BIK	TATGGCTCTGCAATTGTCACC	AGTAGATTCTTTGCCGAGG
BAX	AACTGATCAGAACCATCATG	AGATGGTCACGGTCTGCCACG
NOXA	CCAGTAAATCAGTAGACTGA	ATCTTATCAAGTTACTTCTG
TGFB1	TGGAGCCGCTGCCCATCGT	GGACCTCAGCTGCACTTGCA
HMOX2	ACAGCATCCTCTCTATGGG	ACTACCGGAAGCAGCCTAGG
FOXO3a	ATCCAGCCTGACCAACATG	TGGCATGATCTCGGCTCACT
β-Actin	ACTCCATCATGAAGTGTGAC	AGGAGCAATGATCTTGATCT

Supplementary Table-3. Differentially expressed genes in HIPK2-OE cells

Genes	MG63	Saos2	MG63+HIPK2	Saos2+HIPK2
TGFB1	8.7	10.2	-6.8	-7.8
IL1B	8.3	9.7	-6.5	-9.2
TNFA	7.6	6.8	-5.8	-4.5
ABCCB6	7.2	7.3	-5.4	-6.5
SGSM3	6.7	8.2	-6.1	-4.3
HMOX2	6.4	5.6	-4.5	-6.7
ABCB2	5.8	7.2	-5.6	-3.5
IFNA1	5.5	5.1	-6.8	-6.5
CDH1	5.2	4.6	-4.3	-2.4
ZHX2	4.6	4.3	-3.5	-5.6
ARRB2	4.2	6.7	-6.5	-6.2
GSTA2	3.7	4.7	-4.4	-4.4
CBX4	3.5	5.4	-2.6	-2.3
NRIP1	3.2	4.3	-5.6	-5.4
CUX1	2.5	2.6	-3.2	-6.5
MBD2	2.2	3.4	-4.3	-3.6
HIPK2	-12.1	-10.4	6.3	4.5
BIM	-10.4	-8.9	5.6	7.2
BAX	-9.4	-7.5	5.1	5.4
BIK	-8.3	-6.5	3.4	6.7
NOXA	-8.1	-8.2	5.4	4.3
XPC	-7.6	-5.6	3.2	5.6
STUB1	-6.7	-4.5	4.3	2.3
PUMA	-6.2	-6.7	2.1	4.3
CDR1	-5.4	-4.2	5.6	4.6
PERP	-5.3	-5.6	3.4	4.1
CDKN1A	-5.2	-3.2	2.7	5.4
PDCD4	-4.3	-5.1	4.6	3.2
